# Undifferentiated pleomorphic sarcoma of the small intestine presenting with intussusception in a patient with prior well-differentiated liposarcoma

**DOI:** 10.1093/jscr/rjag724

**Published:** 2026-08-21

**Authors:** Shu Otsu, Yoshinori Murao, Hideto Shiraki, Ayano Tsutsumi, Yoshitake Endo, Shin-ich Nakatsuka

**Affiliations:** Department of Surgery, Yao Tokushukai General Hospital, 1-17 Wakakusa-cho, Yao, Osaka 581-0011, Japan; Department of Emergency Medicine, Yao Tokushukai General Hospital, 1-17 Wakakusa-cho, Yao, Osaka 581-0011, Japan; Department of Surgery, Yao Tokushukai General Hospital, 1-17 Wakakusa-cho, Yao, Osaka 581-0011, Japan; Department of Surgery, Yao Tokushukai General Hospital, 1-17 Wakakusa-cho, Yao, Osaka 581-0011, Japan; Department of Surgery, Yao Tokushukai General Hospital, 1-17 Wakakusa-cho, Yao, Osaka 581-0011, Japan; Department of Pathology, Yao Tokushukai General Hospital, 1-17 Wakakusa-cho, Yao, Osaka 581-0011, Japan

**Keywords:** small intestine tumor, intussusception, undifferentiated pleomorphic sarcoma

## Abstract

An 88-year-old man presented with abdominal pain and vomiting. Contrast-enhanced computed tomography revealed a target sign, suggesting intussusception caused by a small-bowel tumor. Laparoscopic exploration was performed, followed by manual reduction using the Hutchinson technique and segmental small-bowel resection. Histopathological examination revealed a 3-cm submucosal tumor composed of pleomorphic tumor cells. Immunohistochemistry showed no specific lineage differentiation, and staining for MDM2 and CDK4 was negative. Because of his history of well-differentiated liposarcoma, metastatic disease, and dedifferentiated liposarcoma were considered. However, re-evaluation of the previous specimen showed no histological similarity to the present tumor, and the tumor was diagnosed as primary undifferentiated pleomorphic sarcoma of the small intestine. The postoperative course was uneventful, with no evidence of recurrence at the 2-year follow-up. Histological and immunohistochemical comparison is important in patients with a history of well-differentiated liposarcoma.

## Introduction

Adult intussusception is relatively rare and is often caused by an underlying neoplastic lesion [1]. Primary tumors of the small intestine are also uncommon, and preoperative diagnosis is frequently difficult. We report a case of small-bowel sarcoma presenting with intussusception. Because the patient had a history of well-differentiated liposarcoma, it was necessary to distinguish a primary small-bowel tumor from metastatic disease or dedifferentiated liposarcoma.

## Case report

An 88-year-old man presented with vomiting and abdominal pain. His medical history included gastric cancer treated with laparoscopic distal gastrectomy, well-differentiated liposarcoma of the medial aspect of the left thigh that had been resected approximately 2 years earlier and had recurred locally at the same site, hypertension, and prostate cancer treated with brachytherapy.

He developed abdominal pain and vomiting after lunch on the day before presentation. Because the symptoms persisted, he presented to our hospital and was referred to the Department of Surgery with suspected bowel obstruction. His abdomen was distended, with mild epigastric tenderness. Laboratory tests showed no significant abnormalities, and tumor marker levels were within normal ranges.

**Figure 1:**
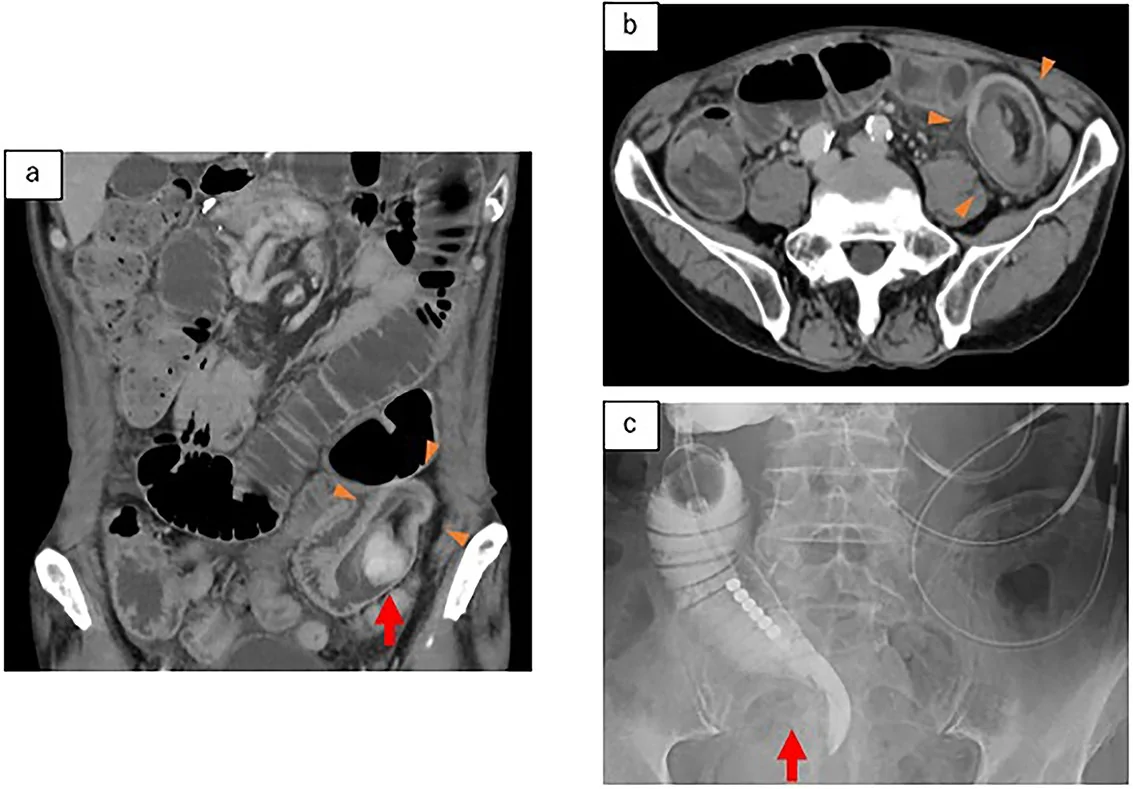
Contrast-enhanced abdominal CT images showed intussusception caused by an enhancing tumor serving as the lead point, with a target sign (arrowheads): (a) coronal view and (b) axial view. (c) A contrast study through a long intestinal tube showed a crab-claw appearance, with no distal passage of contrast medium (arrow).

Contrast-enhanced computed tomography (CT) showed marked small-bowel dilation and a target sign in the pelvic small intestine, suggesting intussusception caused by an enhancing small-bowel mass serving as the lead point (Fig. 1a and b). A contrast study through a long intestinal tube showed a crab-claw appearance, with no distal passage of contrast medium (Fig. 1c). Based on these findings, a diagnosis of intussusception caused by a small-bowel tumor was made, and surgery was performed.

Laparoscopic exploration revealed an intussuscepted segment of small intestine in the left lower abdomen. Because laparoscopic reduction was difficult, the umbilical incision was extended, and the intussusception was manually reduced using the Hutchinson technique. Approximately 40 cm of the small intestine was invaginated at a site 220 cm distal to the ligament of Treitz. Segmental resection of the small intestine was performed with a 10-cm margin from the tumor, followed by functional end-to-end anastomosis. The surgical margins were negative, indicating R0 resection.

**Figure 2:**
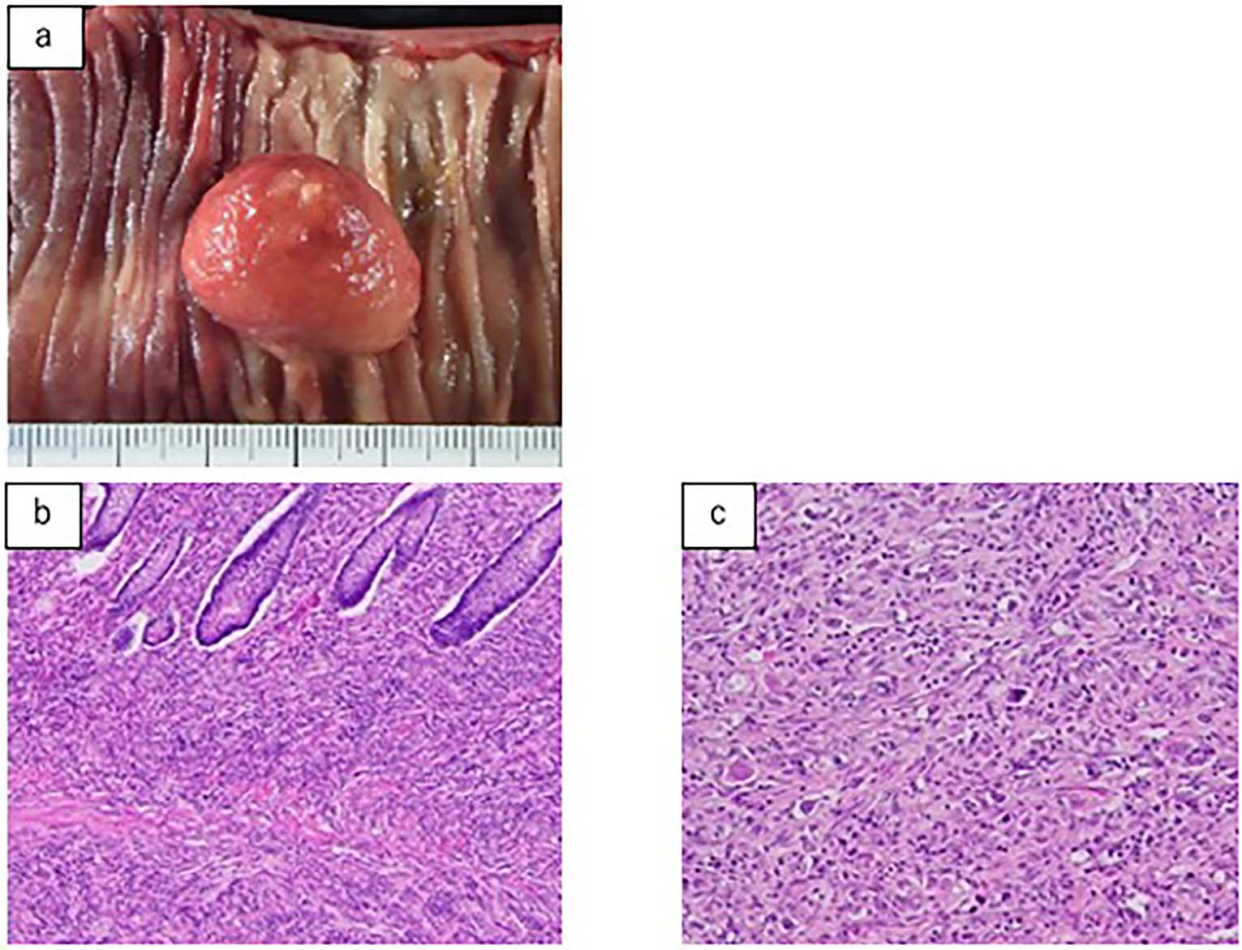
(a) The resected specimen showed a mass measuring approximately 3 cm in the small intestine. (b, c) Histopathological examination showed proliferation of tumor cells with large pleomorphic nuclei and prominent nucleoli. No adipocytic component or lipoblasts were identified. Hematoxylin and eosin staining; original magnification, ×100 (b) and ×200 (c).

The resected specimen showed a protruding mass measuring 30 × 20 × 20 mm (Fig. 2a). The tumor was mainly located in the submucosa and had invaded almost the entire bowel wall. Histopathological examination revealed proliferation of tumor cells with large pleomorphic nuclei and prominent nucleoli, accompanied by inflammatory cell infiltration (Fig. 2b and c). No adipocytic component or lipoblasts were identified.

**Figure 3:**
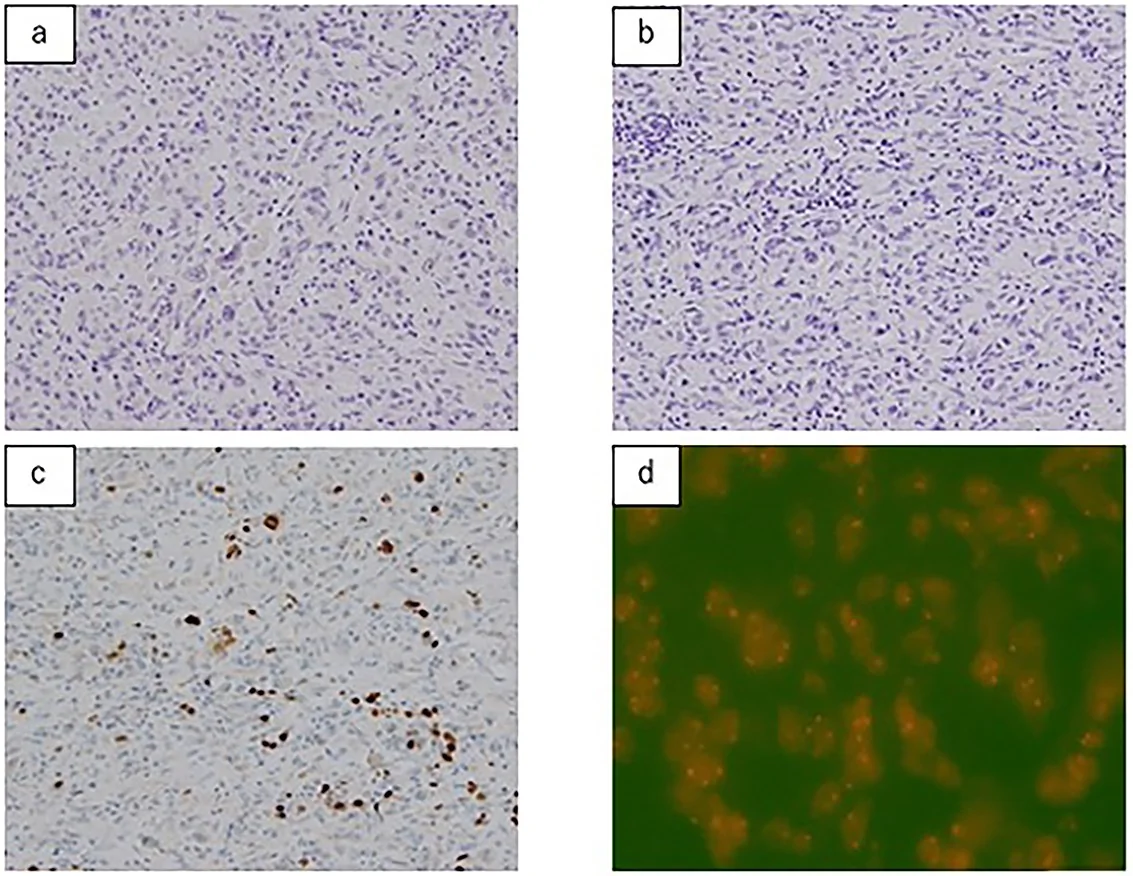
Immunohistochemical and molecular findings. (a) MDM2 staining was negative. Original magnification, ×200. (b) CDK4 staining was negative. Original magnification, ×200. (c) The Ki-67 labeling index was approximately15%. Original magnification, ×200. (d) Fluorescence in situ hybridization for MDM2 showed no MDM2 gene amplification.

Immunohistochemical staining was negative for cytokeratin AE1/AE3, CAM5.2, CD20, CD3, smooth muscle actin, S-100, c-kit, CD34, MDM2, and CDK4. MDM2 gene amplification was not detected by fluorescence in situ hybridization, a finding that argued against dedifferentiated liposarcoma (Fig. 3a, b, and d). The Ki-67 labeling index was approximately 15% (Fig. 3c). The tumor was therefore diagnosed as undifferentiated pleomorphic sarcoma of the small intestine. Re-evaluation of the previous thigh liposarcoma specimen showed findings consistent with well-differentiated liposarcoma and no histological similarity to the present tumor.

The postoperative course was uneventful, and the patient was discharged on postoperative day 9. He has remained free of recurrence for 2 years after surgery.

## Discussion

Adult intussusception is rare, accounting for approximately 1% of all cases of intussusception, and differs from pediatric intussusception because most adult cases have an organic cause, such as a neoplastic lesion, inflammatory disease, or adhesions [1]. Because malignant tumors are frequently involved in adult intussusception, surgical resection is generally recommended. Preoperative diagnosis is often difficult. In the present case, contrast-enhanced CT showed intussusception caused by a small intestinal tumor serving as the lead point, and prompt surgery was performed.

The differential diagnosis of mesenchymal tumors of the small intestine includes gastrointestinal stromal tumor, leiomyosarcoma, schwannoma, and other sarcomas. Undifferentiated pleomorphic sarcoma (UPS) is a high-grade undifferentiated sarcoma without specific differentiation or a specific diagnostic marker. Therefore, UPS is diagnosed by excluding other tumors based on histopathological and immunohistochemical findings. UPS corresponds to tumors previously classified as malignant fibrous histiocytoma and has been redefined in the World Health Organization classification [2].

In the present case, the main diagnostic issue was distinguishing primary UPS of the small intestine from metastatic disease or dedifferentiated liposarcoma related to the previous liposarcoma. Dedifferentiated liposarcoma can arise from well-differentiated liposarcoma and may show UPS-like morphology, making morphological distinction difficult [3, 4]. Therefore, histological comparison with the previous tumor and assessment of MDM2 and CDK4 expression and MDM2 gene amplification are important. In particular, MDM2 amplification supports the diagnosis of dedifferentiated liposarcoma and is useful for distinguishing it from UPS [3].

In this case, immunohistochemical staining was negative for MDM2 and CDK4, and MDM2 gene amplification was not detected by fluorescence in situ hybridization. No adipocytic component, lipoblasts, or transition from a well-differentiated liposarcoma component was identified. In addition, re-evaluation of the previous thigh liposarcoma specimen showed findings consistent with well-differentiated liposarcoma and no histological similarity to the present small intestinal tumor. Although the previous liposarcoma had recurred locally, the recurrent lesion had remained stable in size. These findings supported the diagnosis of primary UPS of the small intestine rather than metastatic disease or dedifferentiated liposarcoma.

Primary UPS of the small intestine is very rare. A search of PubMed and Ichushi-Web using the keywords ‘small intestinal tumor,’ ‘undifferentiated pleomorphic sarcoma,’ and ‘malignant fibrous histiocytoma’ identified nine cases, including the present case, reported between 1992 and 2025 after excluding conference abstracts [5–12] (Table 1). The reported cases presented mainly with abdominal pain, obstruction, bleeding, or systemic symptoms, with variable outcomes. The present patient has remained recurrence-free for 2 years, although careful follow-up is required.

**Table 1:** Previously reported cases of primary undifferentiated pleomorphic sarcoma of the small intestine.

No.	Author	Year	Age	Sex	Symptom	Tumor diameter (cm)	Treatment	Outcome	Other distant metastasis
1	Honda M [5]	1993	76	M	Abdominal pain	14	Small bowel resection	2-years post-operative survival	Peritoneal
2	Chuma M [6]	1997	70	M	General fatigue	8.5	None	Death 2 months after diagnosis	Not described
3	Hanaoka T [7]	2000	45	F	General fatigue	7	Small bowel resection	Survival	Not described
4	Fu DL [8]	2007	43	M	Intussusception	8	Small bowel resection	Death 2 months postoperatively	Lung
5	Katsourakis A [9]	2011	67	F	Weight loss	6.4	Small bowel resection + Chemotherapy	Death 2 months postoperatively	Lung
6	Saneesh PS [10]	2020	29	M	Intussusception	7.5	Small bowel resection	Unknown	Lung/Bone
7	Song SO [11]	2021	60	M	Intussusception	4.6	Small bowel resection	Unknown	Bronchus
8	Gowda AM [12]	2025	40	M	Abdominal pain	12	Right hemicolectomy + Chemotherapy	Survival	Not described
9	Our case	2026	88	M	Intussusception	3	Small bowel resection	Survival	Not described

When a small intestinal sarcoma occurs in a patient with a history of liposarcoma, metastatic disease, and dedifferentiation should be considered. Integrated assessment of the clinical course, histological comparison with previous specimens, and immunohistochemical and molecular findings, particularly MDM2 and CDK4 status, is important for accurate diagnosis.
